# Estonian adverse events study for multimorbid patients using Estonian Trigger Tool (MUPETT—MUltimorbid Patients—Estonian Trigger Tool). Development of Estonian trigger tool for multimorbid patients. A study protocol for mixed-methods study

**DOI:** 10.1371/journal.pone.0280200

**Published:** 2023-03-16

**Authors:** Angela Kannukene, Carola Orrego, Margus Lember, Anneli Uusküla, Kaja Põlluste

**Affiliations:** 1 Department of Medicine, Institute of Clinical Medicine, University of Tartu, Tartu, Estonia; 2 Avedis Donabedian Research Institute (FAD), Universitat Autònoma de Barcelona, Barcelona, Spain; 3 Clinic of Internal Medicine, Tartu University Hospital, Tartu, Estonia; 4 Department of Medicine, Institute of Family Medicine and Public Health, University of Tartu, Tartu, Estonia; 5 Clinic of Dermatology, Tartu Univeristy Hospital, Tartu, Estonia; Public Library of Science, UNITED STATES

## Abstract

**Introduction:**

It is widely recognized that providing healthcare may produce harm to the patient. Different approaches have been developed to measure the burden of adverse events (AEs) to plan and measure the effects of interventions. One of the most widely used instruments is the Trigger Tool, which has previously been modified to be used on various settings and translated into many languages.

Multimorbidity complicates care and may increase the number of AEs patients experience. Currently there is no instrument designed to measure AEs in multimorbid patients. In Estonia, there is currently no validated instrument to measure the burden of AEs.

**Aims:**

The aim of this study will be evaluating the characteristics and ocurrence of AEs in multimorbid patients in hospitalised internal medicine patients of Estonia, and describes the development of a trigger tool for this purpose.

**Methods and analysis:**

We will search for the evidence on measuring AEs in the population of multimorbid patients focusing on trigger tools, and synthesize the data. Data collection of the triggers from the literature will be followed by translating triggers from English to Estonian. An expert multidisciplinary panel will select the suitable triggers for this population. Trigger tool will be pre-tested to assess agreement among professionals and usability of the tool. Validation will be done using 90 medical records. A cross-sectional study in internal medicine departments of two Estonian tertiary care hospitals will be performed to identify the frequency and characteristics of AEs in 960 medical records. We will also provide preventability potential and influencing factors.

**Dissemination:**

Results will be disseminated to healthcare providers and stakeholders at national and international conferences, and as a doctoral medical thesis.

## Introduction

Patient safety is a cornerstone of quality in health care. Since the report To Err is Human [1] was published in 2000, it has been widely recognized that the healthcare delivery may produce harm to the patient. The aim of patient safety is to prevent and reduce the risks that are associated with health care delivery.

One of the most effective ways to make healthcare delivery safer and reduce costs associated with healthcare delivery is reducing the frequency of adverse events (AE). AE is defined as an unintended injury or complication, resulting in prolonged hospital stay, disability at the time of discharge or death, and caused by healthcare management rather than by the patient’s underlying disease process [2]. AEs are common: it is estimated that 10% of in-hospital stays have an AE and about half of them are preventable [2,3]. The impact of AEs from the economic perspective is also high. Treatment of patients with AEs has been estimated to be more costly than patients without one [4].

Patients with multiple chronic illnesses are common, especially in the field of internal medicine. Multimorbid patients (usually defined as patients with at least 2 chronic conditions [5]) have a greater burden of medications, and their treatment is more complex, which may cause bigger incidence of AEs. Multimorbid patients have longer hospital stays [6]; more conditions are correlated with more hospitalizations and higher morbidity [7]. In Estonia, a third of the population has a multimorbid conditions and the burden of multimorbidity increases with age [8]. Currently, Estonia has no validated instrument in use to measure the incidence and characteristics of adverse events consistently and reliably. To effectively plan interventions to reduce the burden of adverse events and measure these interventions, having an effective measurement instrument is essential.

In a population of elderly and multimorbid medical patients, the most common AEs have been described to be healthcare associated infections, followed by neurological reactions (such as delirium), readmissions and skin/tissue damage [9].

In the geriatric population, younger patients, those who have more medications, don’t have surgical operations, have longer stays or more admissions, have more adverse drug events [10]. Most common drugs associated with adverse drug events in elderly comorbid patients were corticosteroids, loop diuretics, opioid analgesics and oral anticoagulants [11]. In elderly patients the incidence of AEs and preventable AEs is higher compared to non-elderly patients, the difference comes from the higher incidence of AEs with medical procedures, preventable drug AEs and preventable falls [12].

As there is little evidence in the literature about AEs in the multimorbid population, there is a need to develop a tool which evaluates the incidence and characteristics of AEs in multimorbid patients with the aim to direct interventions to reduce AEs in this population.

### a) Measuring adverse effects in health care

Adverse events can be detected by different methods. Voluntary reporting systems rely on people willingly submitting information, with this method adverse events are underreported [13]. Manual retrospective chart review, another approach for detecting AE, has been considered the gold standard for determining the frequency of AEs, but this method is time consuming and costly.

Examining charts by preselected signs (triggers) that could indicate the occurrence of AE can make chart review more feasible. Triggers are clues (for example an abnormal laboratory value or a blood transfusion) that are used to select patient charts for further review to determine whether an adverse event has happened. A systematic review by Hibbert et al in 2016 [13] concluded that in a general hospital 30–35% of all admissions have an AE and that voluntary incidence reporting finds about 4% of AEs compared to using the trigger based approach.

Institute for Healthcare Improvement (IHI) global trigger tool (GTT) [14] uses two-stage review process, first stage to find out potential cases with AEs, then second stage to determine whether an AE had happened. A systematic review has been conducted to assess the reliability and validity of the GTT [15], which found that GTT is reliable (percentage of agreement for reviewers being 83–94%) and mean Cohen’s kappa for inter-rater reliability between studies was 0.65, showing that overall inter-rater reliability is substantial. This study however found no studies in which validity of the GTT had been studied. The reliability of GTT in the context of being a standalone outcome measure has been questioned, as the test-retest reliability has been found to be poor by one study [16].

### b) Use of trigger tools in different settings

Trigger tool-based methods have been adapted to many clinical contexts and translated to different languages. There are modified trigger tools for emergency department [17], surgery [18], paediatrics [19,20], geriatric patients [10], home healthcare [21,22], and for discovering medication errors [11]. GTT has been translated and modified to be used in different countries, for example Italy [23], Brazil [24], Portugal [25], China [10], Germany [18], Switzerland [9], Finland [26], Sweden [27], Norway [28] and Denmark [16].

The aim of this study is to indentify and characerize adverse events in hospitalised multimorbid patients in internal medicine departments of Estonia, and develop a trigger tool intended to be used on this population.

## Methods and design

Study design: Mixed methods study, study years 2022–2025.

### Developing MUPETT–description of trigger tool methodology

MUPETT is a method of retrospective 2-stage chart review.

The review team is composed of individuals with clinical knowledge and expertise on patient clinical documentation: (nurse(s) or pharmacist(s) (n = 2)), and a physician (n = 1) whose role is to authenticate the findings and the severity rating of the AEs.

**a) Chart review process.** In the first step, records are reviewed by nurses for the triggers with a limit of 20 minutes for each record. A positive trigger does not necessarily mean that an adverse event has occurred: a focused review in the second step by a physician will determine whether an AE has occurred; a physician will also classify adverse events by the severity of damage.

#### Definitions of the outcomes

An adverse event is determined to be present when there is unintentional harm associated with delivery of healthcare (rather than the progression of the disease). Harm is defined as an additional intervention, monitoring, use of resources, patient disability (either temporary or permanent) or death. If a medical error has happened, but there is no harm, then it is not considered an adverse event. We only evaluate physical harm in this study.

Positive trigger is a trigger that is found from the chart.

Level of harm following an AE will be determined by a reviewing physician and will be categorized as follows (according to National Coordinating Council for Medication Error Reporting and Prevention (NCC MERP) [29]): temporary harm requiring intervention (E), temporary harm requiring prolonged hospitalization (F), permanent harm (G), interventions required to sustain life (H), and death (I).

Preventable AE is considered as the harm to patient which has happened due to an error of delivering health care. An error is defined as a failure to carry out a planned action as intended or application of incorrect plan [30].The preventability is evaluated in a 6-point scale, with 1 being completely avoidable and 6 being completely unavoidable. An AE is considered avoidable when it has been rated 1–3 by reviewer’s opinion.

Multimorbidity is defined as the coexistence of two or more chronic diseases in the same individual. [31] In the purpose of this study, new diagnosis of a disease which may become chronic (e.g., new diagnosis of diabetes or first episode of depression) is not considered as a chronic illness, even if this the cause of hospitalisation.

Time of the adverse event: before, during, or after the index admission. Index admission the admission from which the chart is obtained from. We include all adverse events that have happened during, 30 days before and 30 days after the index admission.

This study has received ethics committee approval (352T-13) by Research Ethics Committee of the University of Tartu. From the participants of the expert group and professionals who are in the pre-test group, written informed consent will be obtained. For the retrospective chart review the patients informed consent will not be obtained.

**b) Target population.** The Estonian Trigger Tool for Multimorbid patients (MUPETT) is intended to be used on adult (at least 18-year-old) acute care patients of internal medicine departments who have been hospitalized for at least 24 hours and who have at least two chronic conditions. Following patients are excluded: patients who have been hospitalized <24 hours, paediatric patients, ambulatory patients and emergency medicine patients, patients in surgical and psychiatric units and units specialized only on intensive care. MUPETT is intended to be used in acute care hospitals by professionals trained in the trigger tool methodology.
**c) Selection of the adverse effects’ assessment tool.** We have chosen to use the trigger tool to adapt for multimorbid patients in Estonia, as this is one of the methods that assesses the incidence and characteristics of AEs. Compared to manual chart review and Harvard Medical Practice Study this method is less resource intensive and the possibility of automating the search for the triggers could make this method more accessible for more healthcare institutions.
**d) Data management, sharing and dissemination.** We will share the information about the study with the patients’ council of the Hospital of University of Tartu. We especially focus on the approach to the adverse events, the background and necessity of the study. After the study we will have another meeting with the patients’ council to let them know about the results of the study. They will have the opportunity to share their opinions.

We can’t involve patients in the validation process, because finding and deciding about adverse events requires specific skills that only experts in healthcare can provide.

Information collected from the patient charts will be stored in password-protected server (only authors of this paper have the access to the data.

Data sets of collected triggers from the literature will be available to researchers interested in testing other hypotheses with the study’s results. Data that includes information from patient charts will be shared generalized and anonynomised. Results from the study will be disseminated at national and international conferences, and as a doctoral medical thesis.

### Development of Estonian trigger tool for multimorbid patients Mixed methods study

**Fig 1:**
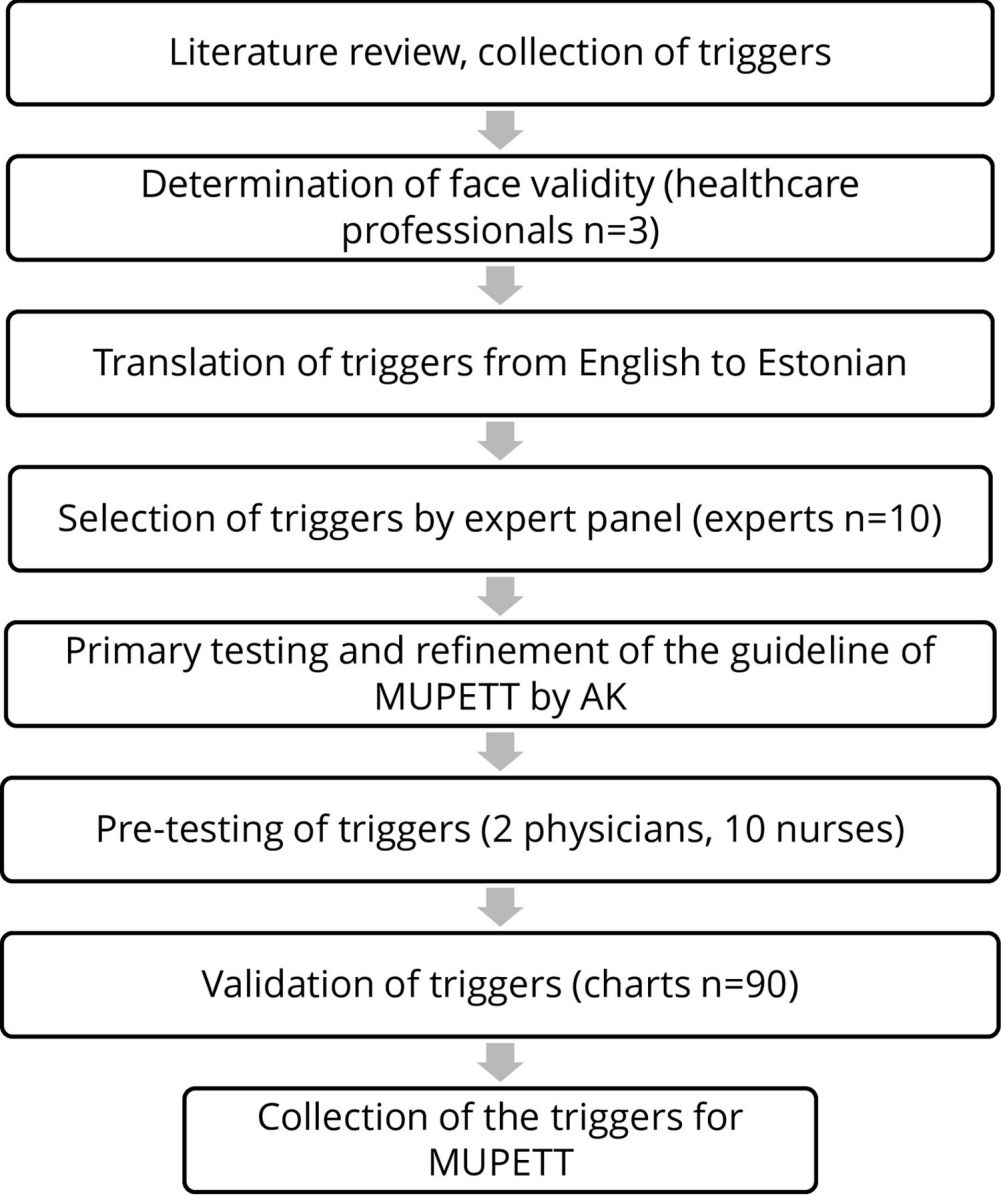
Overview of development of an Estonian trigger tool to evaluate adverse events in multimorbid patients. MUPETT—MUltimorbid Patients—Estonian Trigger Tool. AK—Angela Kannukene.

Overview of the development of MUPETT is described in Fig 1.

**a) Collection of the triggers: A literature review** To find possibly relevant triggers we will search MEDLINE and Scopus libraries for different versions and translation protocols of the trigger tool. We will only include articles published in English. We will exclude emergency medicine, psychiatric, ambulatory, pediatric, perinatal and surgical trigger tool versions from our study, as these are not relevant for the study population. We will not exclude trigger tools intended to be used on intensive care patients, as in Estonia, internal medicine units include lower acuity intensive medicine beds (including vasoactive treatment, high-flow nasal cannula, non-invasive ventilation, mild sedation, but excluding continuous dialysis and intubation). We will make a collection of triggers from these papers, excluding duplicate triggers.
**b) Determining the face validity.** After the collection of the triggers, we will determine the face validity of collected triggers: three healthcare specialists who are familiar with Estonian healthcare systems and multimorbid patients will independently review the list of triggers and exclude triggers that are clearly not relevant for multimorbid patients or for Estonian context. The lists are compared and triggers that have been evaluated to be irrelevant by at least two of the experts will be excluded.
**c) Translation and validation of triggers.** Collected triggers will be translated from English into Estonian.

Translation from English to Estonian by two independent bilingual translators, one of whom is an expert in English language and the other is expert in patient safety. After translation, a third person who is familiar with the Estonian internal medicine patients and fluent in English, reviews the translations and decides on the best translation. A meeting will be held to discuss discrepancies and a consensus will be sought.

**d) Selection of triggers by an expert panel.** The expert panel will consist of professionals from relevant disciplines: At least two internal medicine physicians (one with subspecialty of geriatrics, if possible), one intensivist, infectious disease doctor, at least four nurses (working with hospitalized internal medicine patients), and a pharmacologist. An informed consent will be obtained from the participants. Triggers will be sent to participants before the meeting so to determine their decision of best triggers before meeting.

The meeting will be moderated by one of the authors (AK) and observed by another author (KP) of this paper.

For each trigger the applicability and the relevance will be discussed. By consensus of the participants of the expert panel, triggers may be included, removed, or modified. The outcome will be a primary collection of triggers for MUPETT.

**e) Primary testing and refining of the guideline by one of the authors of the paper.** One of the authors (AK) will write a guideline to use MUPETT using previously decided on triggers. Guideline will be tested by AK on 10 charts of multimorbid patients and will be refined to make guideline clearer.
**f) Pre-testing of the MUPETT.** An informed consent will be obtained from the pre-test group participants.

2 physicians and 10 nurses (all experienced in treating multimorbid patients) will receive the same five clinical cases to simulate the trigger tool review process. The review process is described in the section “Methods and design”. The outcomes will be the number and the type of triggers recorded, adverse events identified, and preventability of adverse events found by each professional (as applicable by the role).

Interrater reliability will be estimated using the Fleiss’ Kappa test. We set the threshold of 0.60 as the requirement of the minimal acceptable agreement.

The pre-test group will be asked to rate each item of MUPETT in a 5-point Likert scale (1 –completely disagree, 2 –disagree, 3 –neither disagree nor agree, 4 –agree, and 5 –completely agree) on two statements (easy administration, relevance of integrating it into risk management) to confirm the accuracy and relevance. The content validity coefficient will be calculated for each item. >0.70 will be sufficient agreement for each item.

**g) Validation for MUPETT.** For the validation of the Estonian Trigger tool (index test), we will measure how many of the adverse events are found using Estonian global trigger tool versus retrospective manual full chart review (reference tool) as the gold standard.

We will select 90 applicable random charts from patients discharged from January to April 2021 from internal medicine department. The charts will be selected using a random number generator.

A trigger tool review team will find the triggers from all 90 charts, and AEs from charts that have triggers present using MUPETT methodology and are blinded to the full chart review results. Two physicians blind to the work of the trigger tool team will conduct a full manual retrospective chart review to assess the number of adverse events in all of the 90 charts.

For estimating diagnostic accuracy, we will calculate sensitivity, specificity and accuracy of the MUPETT using manual chart review as a golden standard. Additionally, we will measure precision, positive predictive value (PPV) and negative predictive value (NPV) of MUPETT, PPV and NPV of each individual trigger.

IHI GTT has shown positive predictive value of about 30% (32). A trigger tool based on retrospective cross-sectional study on a random sample of medical records of patients admitted hospital care reported sensitivity of 97.8% (25). The prevalence of admissions having at least one adverse event has been 20–36% [23,25,32], with general medical inpatients having 8–60% admissions with an adverse event (variation in AE rates have been explained by methodological reasons) [9,13]. The PPV cutoff value of 5% for removing triggers from the trigger tool has been used by Hu et al (33).

We will remove the marker from the trigger tool if the PPV is less than 5%. We expect the sensitivity of the MUPETT to be at least 80%.

If the sensitivity of the MUPETT tool in detecting adverse events is less than 80%, we will review the triggers from the expert panel and will include triggers that had the most discussion (but were excluded) to the tool. After the review we will redo the validation process.

### Retrospective cross-sectional multicenter study for describing the burden and characteristics of adverse events in multimorbid patients in Estonia

#### Method

Multicentric retrospective cross-sectional study, using chart review with Estonian trigger tool for multimorbid patients (MUPETT).

#### Setting

Tartu University Hospital and North Estonian Medical Centre.

**a) Study team training.** Review team training will be conducted by the authors of this study (AK and KP). The teams from each hospital will receive a manual for using the MUPETT methodology, which includes the description of the review process, definitions of triggers and adverse events, and specific examples about each trigger. After familiarizing themselves with the manual, study teams will have a training using MUPETT on medical charts.
**b) Study sample, inclusion and exclusion criteria.** Sampling strategy: 20 random charts per month from each calendar year of 2021 and 2022 from each internal medicine department from both hospitals. Multimorbidity will be evaluated by looking through the patients’ diagnosis codes (which include whether it is the first time or repeating diagnosis) and confirmed by reading patients’ history section of the chart.

Inclusion and exclusion criteria have been explained previously in this document.

Sample size: We will sample 480 medical records per site (960 in total).The number of patients for this study was determined by the power calculation: with the sensitivity and specificity of 90% and confidence interval of sensitivity with the precision of +-5%, we need 695 cases if we expect the percentage of adverse events to be 20% per 100 admissions.

Method: We will use Estonian trigger tool for multimorbid patients (MUPETT), translated and validated in the previous part of the study. A trigger tool review team will find the triggers and adverse events using MUPETT, finding positive triggers and adverse events. They will assess the preventability of each adverse event found.

Our outcomes will be positive triggers, adverse events, preventability of adverse events, level of harm caused by AEs, time of the adverse event.

We will collect the following variables: patient’s age, sex, hospital name and unit, length of hospital stay, diagnoses, procedures done, imaging studies, surgeries, result of hospital stay (discharged or died), the date of adverse event, type of adverse event (drug error, procedural, healthcare associated infection, allergic reaction, injury (including falls), equipment or device error, diagnosis error, ward management (including pressure ulcers), type of harm associated with adverse event (intervention, use of additional resources, patient disability or death), total number of discharges and total number of from the internal medicine departments by site.

Statistical methods: We will use descriptive statistics for patient characteristics (age, sex, length of hospital stay, number of diagnoses, number of procedures, result of hospital stay, preventability): mean and standard deviation, median, percentages where applicable. We will calculate adverse events per 1000 patient days, adverse events per 100 admissions and percent of admissions with an adverse event, prevalence of AEs.

## Discussion

We have presented the method and expected outcome of developing and using a trigger tool for finding adverse events in a population of multimorbid patients in the setting of general internal medicine. We describe the collection of the triggers from the literature, process of the selection of the triggers, validation, and testing of Estonian trigger tool. Furthermore, we will describe the process we will use for our study to characterize the adverse events in Estonian multimorbid patients in two academic hospitals.

The main outcome of this study will be the developed and validated trigger tool for multimorbid internal medicine patients. As there is no trigger tool developed for multimorbid people internationally, there is a possibility to translate and validate the trigger tool for other countries In Estonia, this provides the first insight of the characteristics and preventability of adverse events in Estonian context.

Furthermore, this study will allow us to obtain the data to focus on what kind of interventions need to be planned to reduce the number of adverse events. The experience and data collected may allow us to modify and expand the trigger tool in Estonia from the setting of inpatient medical patients to other areas as well, for example, multimorbid patients in surgery or ambulatory care.

This study will allow us to compare our results to results presented in literature. Previous studies in hospital settings have estimated that 10% of the stays have an AE [2], with about half of them being preventable [2]. Similarly as reported in literature in other hospital populations, we expect our most common AEs to be infections, neurological reactions, readmission within 30 days and skin/tissue damage [9].

For the clinical practice this study will provide the knowledge about the situation of adverse events in Estonian patients; clinicians may be able to notice and prevent some of the adverse events if they know the risk areas. This study will allow us to engage medical professionals to notice and improve the areas of patient safety. Hospital managers can use the collected data to prioritize interventions to reduce the adverse events in this population. As we introduce the method of trigger tools into Estonia, there is a future chance to implement and expand the use of trigger tools for other areas of medicine, for example surgical units or ambulatory units. In the future, the possibility of automatizing trigger tool would make wider implementation of trigger tool in Estonian hospitals less resource intensive.

In future scientific research, there is a need to identify adverse events of multimorbid patients in different countries and other types of hospitals. The modification and validation of trigger tools for this population will help to identify the effect of intervention methods for reducing the number of adverse events.

This study and sharing of information regarding adverse events to the general public provides an opportunity to educate patients and clinicians about the factors and causes of adverse events, as well as assist alter the culture to one that is blame-free and open to dialogue.

The limitation of this study is that the data that will be collected is from purposive sample of hospitals (only 2 tertiary care centers from Estonia will be included). This means that these results are not generalizable to all hospitals in the country. The use of medical records carries an inherent risk that there are adverse events that are not documented and therefore cannot be found by retrospective review.

Regardless, this study will provide the first insight into AEs in multimorbid patients, and would provide experience and insight to expand studying AEs in other settings and populations in Estonia.

## Supporting information

S1 Checklist STARD (Standards for Reporting of Diagnostic Accuracy Studies) checklist completed for the article. N/A–not applicable.
(PDF)

S2 Checklist STROBE (STrengthening the Reporting of OBservational studies in Epidemiology) checklist completed for the article. N/A–not applicable.
(PDF)
